# Interventions to enhance adherence to dietary advice for preventing and managing chronic diseases in adults: a study protocol

**DOI:** 10.1186/1471-2458-11-111

**Published:** 2011-02-17

**Authors:** Sophie Desroches, Annie Lapointe, Stéphane Ratté, Karine Gravel, France Légaré, Jayne Thirsk

**Affiliations:** 1 CHUQ Research Center (Centre Hospitalier Universitaire de Québec-Hôpital St-François-d'Assise), Québec, Canada; 2 Department of Food and Nutrition Sciences, Faculty of Agriculture and Food Sciences, Laval University, Québec, Canada; 3 Department of Family and Emergency Medicine, Faculty of Medicine, Laval University, Québec, Canada; 4 Practice Based Evidence in Nutrition, Dietitians of Canada

## Abstract

**Background:**

Adoption of a healthy diet has been identified as the cornerstone in the prevention and management of chronic diseases. However, non-adherence to lifestyle changes raises an important issue since adherence level to dietary advice is a key determinant of the effectiveness of dietary treatment. Therefore, based on the results of a Cochrane systematic review on interventions for enhancing adherence to dietary advice for preventing and managing chronic diseases in adults, the aim of this study is to assess the importance and applicability of interventions enhancing adherence to dietary advice in the Canadian context.

**Methods/Design:**

In phase 1, dietitians' opinion will be assessed through a Delphi study regarding the importance and the applicability in the Canadian context of the interventions found the most effective to enhance adherence to dietary advice through a Cochrane systematic review. In phase 2, findings of the Cochrane systematic review assessing the effects of interventions for enhancing adherence to dietary advice will be reported in a practical format on an online knowledge translation tool for dietitians and other health professionals.

**Discussion:**

In recent years, there has been an increasing recognition of the failure to translate research findings into clinical practice. Therefore, knowledge translation efforts need to prioritize effective interventions that will be the most relevant for practice and end-users by adapting them to the local context. Our study will provide decision makers in the field of dietetic practice with essential knowledge on adherence for elaborating educational activities for academic or professional settings that will respond to dietitians' priorities in terms of importance and applicability to day-to-day practice.

## Background

Chronic diseases are the leading cause of death and disability and account for 60% of all deaths worldwide [1]. Several authoritative health agencies have recommended the adoption of a healthy diet as the cornerstone in the prevention and/or management of chronic diseases such as cardiovascular diseases [2], diabetes [3], cancer [4], and hypertension [5]. Although many studies have demonstrated the beneficial impact of dietary treatment in strictly controlled experimental conditions, it remains that its full benefit will only be truly observed in practice settings if patients follow the recommended diet closely. As greater adherence to dietary advice is a critical component for preventing the onset and progression of many diet-related chronic diseases, some studies have developed innovative interventions resulting in a better agreement between evidence-based dietary advice of health professionals and their patients' eating patterns [6,7]. These recent advances developed to improve patient adherence to dietary advice need to be translated to dietitians in their day-to-day practice to produce an optimal effect on population health.

The process of knowledge translation is defined as *a dynamic and iterative process that includes the synthesis, dissemination, exchange and ethically sound application of knowledge to improve the health of Canadians, provide more effective health services and products and strengthen the health care program *[8]. A conceptual framework (knowledge-to-action framework) describing the phases of knowledge translation was developed by Graham and colleagues [9] and integrates the concepts of knowledge creation and knowledge application. Within knowledge creation, evidence-based knowledge is identified by synthesizing studies about a specific clinical question, generally in the form of a systematic review. Then, to facilitate the evidence-based knowledge application into day-to-day practice of health professionals, it also needs to be adapted to the local context [9].

Considering the failure to translate research findings into clinical practice [9] and the importance of preventing and managing chronic diseases and their risk factors in the population [10], this problematic emphasises the importance of critically examining the literature by performing a systematic review with a specific focus on the identification of effective and practical techniques for improving patients' adherence to dietary advice. This, in turn, will provide dietitians and other health professionals with the essential knowledge to dispense optimal care to patients at risk of or with diet-related chronic diseases. Therefore, our aims are to:

1) Assess Dietitians of Canada members' opinion regarding the importance and applicability in the Canadian context of the interventions found to be the most effective to enhance adherence to dietary advice through a Cochrane systematic review (Phase 1);

2) Synthesize findings of a Cochrane systematic review in a practical format for publication on an online knowledge translation tool for dietitians and other health professionals (Phase 2).

## Methods/Design

A full version of the Cochrane systematic review protocol is available in *The Cochrane Library *[11]. This Cochrane systematic review investigates the effects of interventions for enhancing adherence to dietary advice for the prevention and the management of chronic diseases in adults.

### Phase 1: Pan-Canadian Delphi study

In the first phase of the study, findings from the Cochrane systematic review will be validated for their applicability and their importance to dietetics practice in the Canadian context through a Delphi study. The study will involve Dietitians of Canada members with clinical expertise in a variety of populations, health conditions, and practice settings. Some studies have used the Delphi methodology to assess the opinion of dietitians about a specific issue [12-14]. The Delphi study is considered to be a strong methodology for a rigorous consensus of experts on a specific theme. This type of study is highly recommended for obtaining opinions of experts from different regions and settings [15], giving us the opportunity to have a pan-Canadian representation. This phase of our proposal is inspired by work by Grimshaw et al. [16] who previously showed that evidence-based strategies for translating knowledge into clinical practice needs to be balanced by what is felt useful and practical by the target users.

#### Selection and recruitment of the expert panel

A list of potential participants will be created and recruitment of experts will be done through a message sent by email to Dietitians of Canada members identified in the member database as having individual/private counselling skills and expertise in chronic disease management (n = 150), thus ensuring a representative sample and allowing for expected attrition as previously shown in a Delphi study conducted by Maclellan and Berenbaum [12]. A letter of invitation will present the study's objectives and will solicit their participation in the Delphi study. The message will also provide a link to the study website and give participants a coded username and a password to confidentially login. Participants will be informed that their participation to the study is entirely voluntary. Because we will invite experts (dietitians) to give their opinion about the importance and the applicability of the interventions found the most effective to enhance adherence to dietary advice, the Ethics board of the CHUQ-HSFA Research Center considered that the study does not represent any risk for health, dignity or reputation of dietitians and that it is complying with principles that rule Ethics research. Therefore, this study received ethics exemption from the Ethics board of the CHUQ-HSFA Research Center and participants will not have to provide an informed consent.

#### The Delphi process

The first round Delphi questionnaire will be developed from the findings of the Cochrane systematic review. It will present the most effective interventions for improving adherence to dietary advice. The questionnaire will be pilot tested by five dietitians not part of the sample database to ensure clarity and readability of the questions and to evaluate the time needed to complete it. According to their suggestions, the wording and formatting of the questionnaire will be modified by the research team to create a final version of the questionnaire. All potential participants will be sent by email an information sheet about the project. Participants will be guided through the process of the electronic Delphi questionnaire. The first section of the questionnaire will gather socio-demographic information. Participants will then be asked to rate the most effective interventions to enhance adherence to dietary advice on two aspects: 1) priority/importance for dietetics practice in Canada; and 2) applicability to the Canadian context according to their work experience on a seven-point Likert scale. Participants will have the opportunity to comment their responses. Results from the first round will be compiled and a mean score of priority/importance and applicability for each item will be calculated. Then, participants will be invited to participate in a second round rating process by email, through the password protected website. Dietitians will again be asked to rate the degree of priority/importance and applicability of each identified interventions. This survey will also show the first round ratings by providing the dietitian's own rating for each item, the mean score for each item and a summary of the qualitative comments. A third round survey, based on the responses of the second round, might be necessary if a consensus is not reached for at least 70% of items [17]. Finally, the consensual rating will be sent a last time to the experts for a final validation. The quantitative results from the questionnaire will be analysed with SAS (Statistical Analysis System, version 9.2, SAS Institute Inc., Cary, NC) to determine the mean score and the interquartile range for each item. The qualitative comments of the dietitians will be classified into broader themes and the frequency of each theme will be computed with NVivo^© ^software (version 8, QSR International, Melbourne, Australia).

### Phase 2: Synthesis of findings from a Cochrane systematic review adapted for dietitians and other health professionals

In the second phase of the study, results of the Delphi study conducted in phase 1 will be used to further refine the findings from the Cochrane systematic review to publish them in a practical format on the online knowledge translation tool for dietitians and other health professionals: *Practice-based Evidence in Nutrition (PEN) *and *Current Issues, The Inside Story*, both developed by the Dietitians of Canada, the national professional association for dietitians in Canada. *PEN *is an evidence-informed, web-based decision support tool for the dietetic profession and other health professionals while *The Current Issues, The Inside Story *is a monthly service that provides all Dietitians of Canada members with evidence-based timely information on topics of relevance to consumers, the media and the profession.

Results from our Cochrane systematic review will be further summarized, evidence will be graded, and bottom line practice guidance will be provided according to the results of the Delphi study. Links to research abstracts and complete articles, as well as practice tools and resources that are congruent with the evidence, will also be fully integrated into "knowledge pathways" in *PEN*. Once drafted, each knowledge pathway will be peer-reviewed as a further assurance of its scientific integrity before its publication in *PEN*.

Results will also be summarized in a Dietitian of Canada electronic publication, *Current Issues, The Inside Story *which is a monthly service that provides all Dietitians of Canada members with evidence-based timely information on topics of relevance to consumers, the media and the profession. Where appropriate, a consumer information piece designed as an FAQ will be developed to complement the *Current Issues *topic.

## Discussion

In recent years, there has been an increasing recognition of the failure to translate research findings into clinical practice [9]. It is therefore imperative that more research be conducted to identify effective and efficient strategies so that health professionals stay well informed of new, evidence-based research findings through pre-appraised, synthesized evidence, such as the *Cochrane Database of Systematic Reviews *[18]. However, for an effective translation of the research findings into dietitians' or other health professionals' practices, it is insufficient to know that, for example, some interventions are found effective to enhance clients' adherence to dietary advice in research settings. Research needs to also prioritize effective interventions that will be the most implementable and relevant in practice and to adapt them to the local context [16]. The importance of dietitians' involvement in research and knowledge translation remains poorly addressed in the scientific literature [19,20]. Therefore, we strongly believe that our research initiative will favour the development of integrated knowledge translation through a partnership established with the Dietitians of Canada by making available a practical set of evidence-based strategies to enhance patients' adherence to dietary advice and by assessing the importance and applicability of these interventions in the Canadian context. It is expected that this will, in turn, contribute to improving the knowledge base on adherence to dietary advice, a topic of immense importance for dietetics practice that will be relevant to decision makers and end users (e.g. dietitians and patients). Our research project will also provide decision makers with the essential knowledge on adherence for elaborating educational activities for academic or professional settings that will respond to dietitians' priorities in terms of importance and applicability to their day-to-day practice.

## List of abbreviations used

PEN: Practice-based Evidence in Nutrition.

## Competing interests

The authors declare that they have no competing interests.

## Authors' contributions

SD: is the principal investigator, leads the Cochrane systematic review and will be involved in the screening, reviewing, and data extracting; AL: is the project coordinator, and will be involved in the screening, reviewing, data extracting, development of the various tools to be used in the data extraction form, coordination of the writing and publication; SR: provided feedback on the development of the Cochrane systematic review protocol and will design the search strategies; KG: provided feedback on the development of the Cochrane systematic review protocol and will participate in the data extracting; FL: provided expertise in Cochrane systematic review methodology and feedback on the development of the Cochrane systematic review protocol; JT: is the decision maker representing the Dietitians of Canada. She provided feedback on the Cochrane systematic review protocol and will collaborate to the adaptation of results for dietetic practice and to the Delphi study by providing a list of clinical experts, members of the Dietitians of Canada to be contacted. All authors contributed to the writing and editing of the protocol for publication and read and approved the final manuscript.

## Pre-publication history

The pre-publication history for this paper can be accessed here:

http://www.biomedcentral.com/1471-2458/11/111/prepub
